# First person – Ricardo Villalba-Briones

**DOI:** 10.1242/bio.062054

**Published:** 2025-05-21

## Abstract

First Person is a series of interviews with the first authors of a selection of papers published in Biology Open, helping researchers promote themselves alongside their papers. Ricardo Villalba-Briones is first author on ‘ Short-range Bluetooth monitoring method for tracking, monitoring and follow-up of low-mobility wild species, *Choloepus hoffmanni* sp.’, published in BIO. Ricardo conducted the research described in this article while a researcher and lecturer at ESPOL University, Guayaquil, Ecuador, investigating conservation of wild mammals and sustainability. He is now a laboratory technician in water quality at Labaqua, Ibaizabal bidea, Spain.


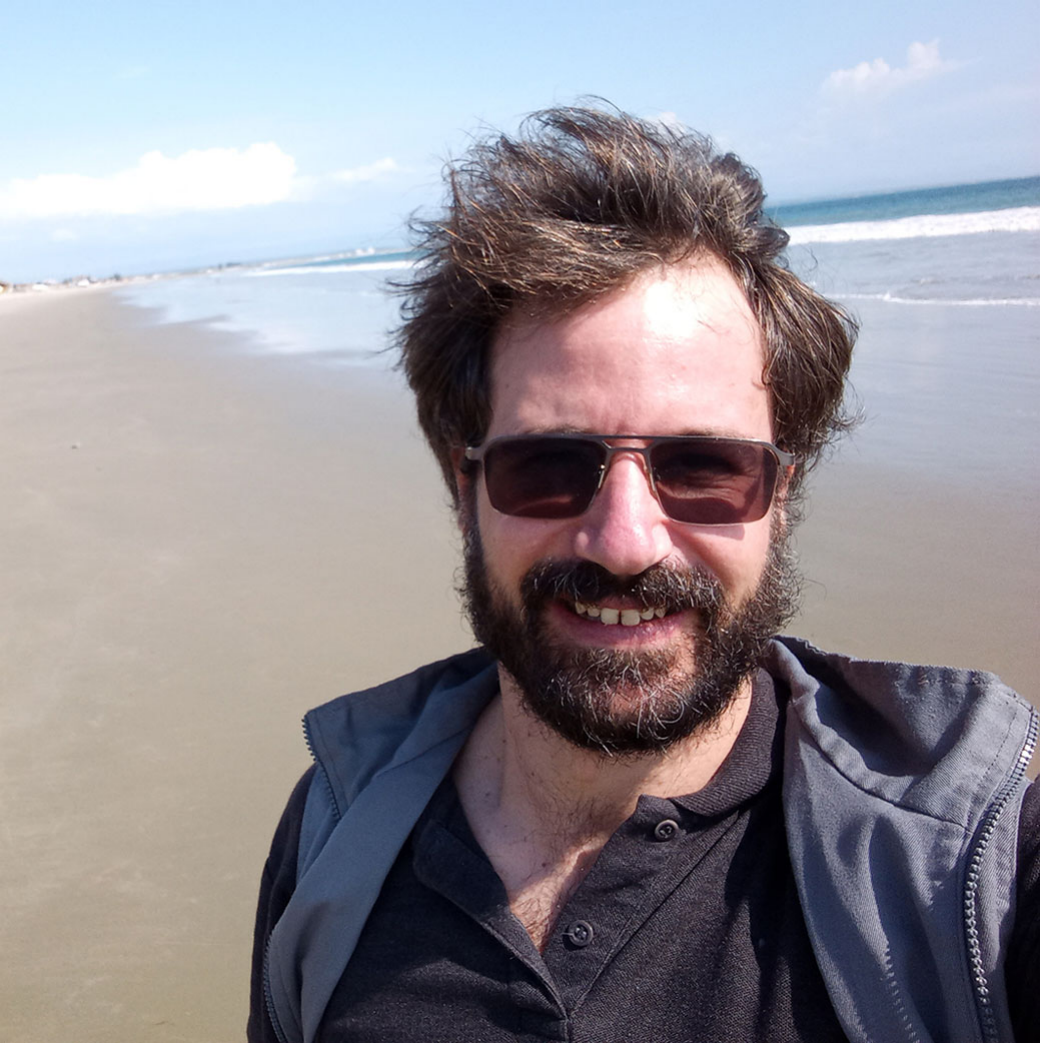


**Ricardo Villalba-Briones**

**Describe your scientific journey and your current research focus**

I began my career by volunteering with non-governmental organizations (NGOs), and, after completing my Master of Science, I had the opportunity to work at ESPOL University. There, I led projects that connected society with academia through conservation initiatives, including the promotion of sustainable dolphin-watching practices. Subsequently, I directed projects focused on community engagement in the conservation of terrestrial mammals, while simultaneously coordinating a program comprising 24 extension-based projects (ESPOL, 2023). My research spans multiple disciplines related to biodiversity conservation, including environmental education, reproduction of Pijio trees, protected area management, wildlife diseases, and the rehabilitation and release of wild mammals.

**Who or what inspired you to become a scientist?**

From an early age, I was captivated by the work of Dian Fossey and Jane Goodall. Their pioneering research on gorillas and chimpanzees, and their commitment to traveling to study these animals in their natural habitats, deeply inspired me. As a teenager, I found very interesting the writings of Richard Dawkins and Desmond Morris, whose books I discovered in my grandfather's personal library. By then I already liked the natural sciences, and I decided to pursue a degree in biology. Over the years, my interest in implementing scientific research gradually grew. Volunteering for the NGO Neotropical Primate Conservation, managed by Sam Shanee and Noga Shanee, was an encouraging experience. The Peruvian rainforest and the fieldwork involved in monitoring woolly monkeys were especially appealing to me. Seeing the diverse initiatives undertaken by this NGO inspired me to keep searching for solutions to environmental challenges. All these experiences made me realize that the biological sciences could offer unparalleled opportunities to address environmental challenges. In the end, my path toward a scientific career was not the result of a single moment of inspiration, but a steady, evolving journey.

**How would you explain the main finding of your paper?**

Wildlife post release can be monitored through a short-range Bluetooth monitoring method (SBMM). Rehabilitation projects have little in the way of funds to achieve their objectives. This method provides a cheap and easy technique to follow-up released animals and to verify their wellbeing in the wilderness. Consequently, animals not fitted for the independent life in their habitat can be managed, rescued or assisted.

This new monitoring method offers a valuable tool for rehabilitation centers to track animals following their release into the wild.

**What are the potential implications of this finding for your field of research?**

This new monitoring method offers a valuable tool for rehabilitation centers to track animals following their release into the wild. The inclusion of a biodegradable component allows the natural detachment and potential recovery of the tracking device. The device costs only $25-30 US, making it accessible to rehabilitation centers where the number of treated animals may be too high to implement more conventional and technologically advanced methods.

**Figure:**
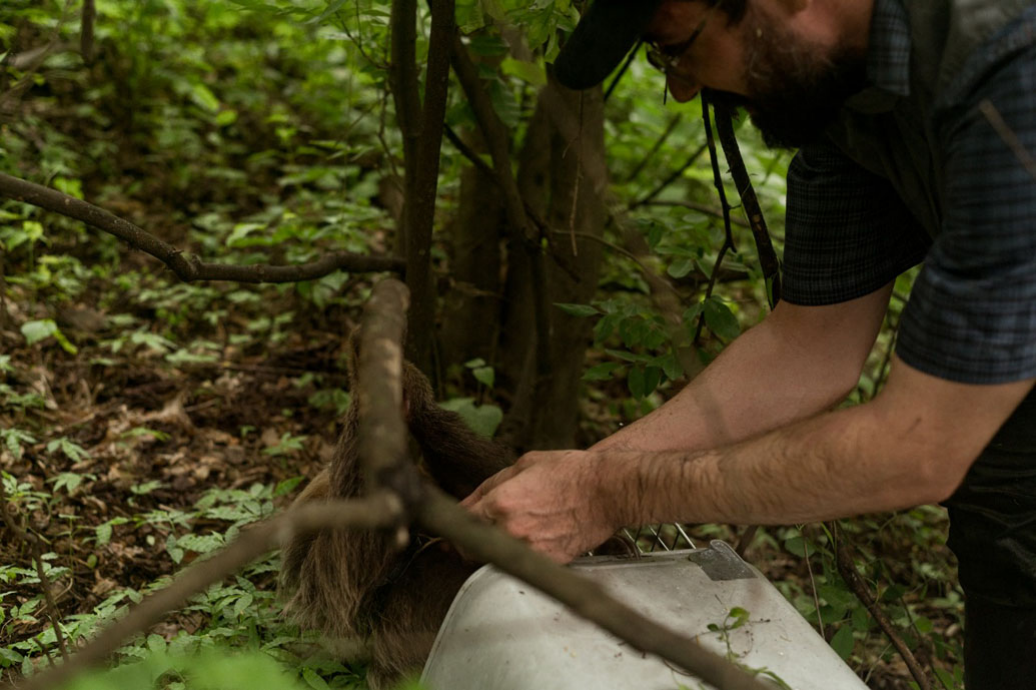
**A rehabilitated two-toed sloth being placed back into its natural habitat.** The tracking backpack attached to its body is checked, ensuring a safe and monitored transition during the reintroduction process.

**Which part of this research project was the most rewarding?**

The most rewarding outcome was observing the two-toed sloths' survival and their display of positive behavioral responses to the environment, such as independent feeding and effective arboreal movement.

**What do you enjoy most about being an early-career researcher?**

Conducting literature research to access relevant information proves to be an invaluable resource for identifying good practices and achieving the project's objectives. The ecological knowledge obtained from scientific articles provides a solid foundation for informed decision making. In my view, this is the area where biologists are best equipped to contribute effectively to the management and care of animals.

Conservation research, particularly in wildlife, remains under-represented and in need of proactive contributions.

**What piece of advice would you give to the next generation of researchers?**

Do not wait for conservation projects to be offered; instead, take initiative by designing and proposing your own research and implementation projects to relevant institutions. Conservation research, particularly in wildlife, remains under-represented and in need of proactive contributions.

**What's next for you?**

I'll teach biology.

**What do you think that biologists can offer to society?**

I believe that the skills and potential contributions of biologists are often underestimated in Western cultures. Although job eligibility varies significantly across countries, it is my perception that, even within conservation management, other professional profiles are frequently prioritized over those with formal training in biology. As I discussed in my thesis (Villalba-Briones, 2024), the discipline of biology is fundamental to addressing the diverse drivers that threaten the wellbeing of species and ecosystems – factors that are inherently tied to sustainability. Therefore, I am confident that, in the future, biological training will be more appropriately valued in positions responsible for maintaining the ecological aspects of productive and sustainable societies. Additionally, I believe that scientific researchers should receive greater institutional support to ensure the continuity and impact of their work.
